# Therapeutic characteristics of patients followed for bipolar disorder with rapid cycles: Study on a Tunisian population

**DOI:** 10.1192/j.eurpsy.2022.1052

**Published:** 2022-09-01

**Authors:** M. Karoui, G. Amri, R. Kammoun, H. Nefzi, F. Ellouz

**Affiliations:** 1 Razi hospital, Psychiatry G Department, Mannouba, Tunisia; 2 Razi Hospital, Manouba, tunis, Tunisia; 3 Razi hospital, Psychiatry G, manouba, Tunisia; 4 Razi hospital, Psychiatry G, denden, Tunisia

**Keywords:** antidepressant, course, rapid cycling, bipolar disorders

## Abstract

**Introduction:**

Bipolar disorder is a frequent and particularly severe psychiatric pathology that causes significant morbidity and mortality. The rapid cycling forms are more severe in terms of their expression, evolutionary course, therapeutic responses and associated comorbidities.

**Objectives:**

The aim of this study is to conduct a descriptive assessment of therapeutic characteristics in patients with rapid cycling bipolar disorder.

**Methods:**

Our work involved a population of 97 patients followed for bipolar disorder diagnosed according to DSM5 criteria, including 37 patients meeting the specification “with rapid cycles”. The patients were divided into two groups: - Group of patients with bipolar disorder with rapid cycles (TBCR) - Group of patients with bipolar disorder without rapid cycling (TBNCR). We compared the therapeutic features among these two groups.

**Results:**

The dominant polarity was depressive in patients with rapid cycles. They required more mood stabilizers. A greater proportion of them had received treatment with serotonin reuptake inhibitor antidepressants. They were more likely to use hypnotics such as antihistamines and zolpidem.

**Conclusions:**

Rapid cycling TB is a relatively common clinical modality that should be investigated and identified.The use of antidepressants is associated with this course of the disease. Their utilization in the treatment of bipolar depression must be thoughtful and well studied

**Disclosure:**

No significant relationships.

